# Supplementary figures and images for: Boosting subdominant neutralizing antibody responses with a computationally designed epitope-focused immunogen
Source: PLoS Biol. 2019 Feb 21;17(2):e3000164. doi: 10.1371/journal.pbio.3000164 (PMC6400402; doi:10.1371/journal.pbio.3000164)

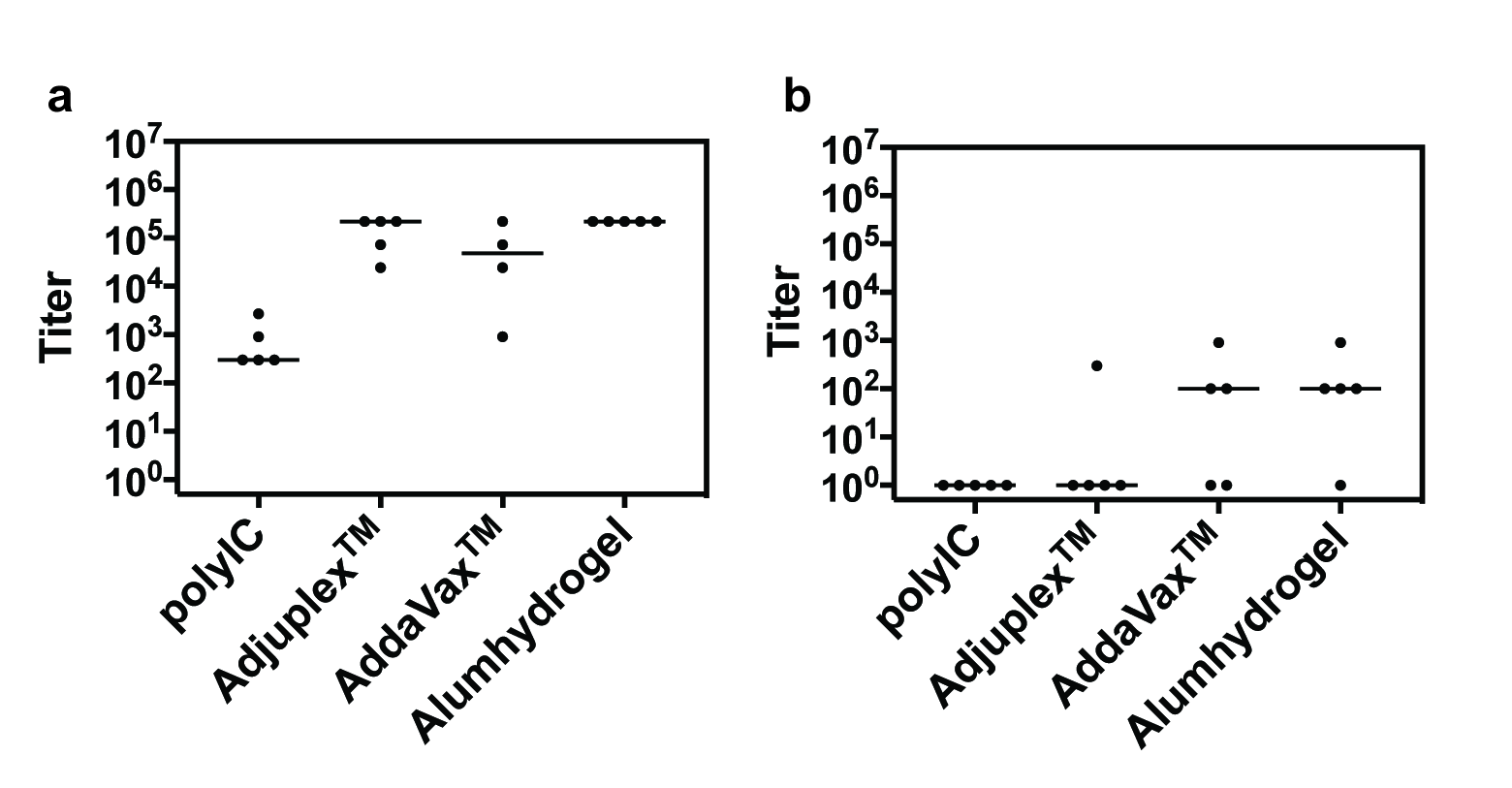

Supplement: S1 Fig — Female Balb/c mice (five animals/group) were immunized three times (days 0, 21, 42) with 10 μg FFL_001 monomer adsorbed to different adjuvants, and serum was analyzed on day 56. (A) Immunogenicity of FFL_001 formulated in different adjuvants. Serum titers were determined against FFL_001 at day 56 of the immunization protocol. FFL_001 adsorbed to alum showed highest overall immunogenicity. (B) Prefusion RSVF cross-reactivity of FFL_001 immunized mice after three immunizations. Four out of five mice immunized with FFL_001 formulated in alum showed serum cross-reactivity with prefusion RSVF. Data are available in S1 Data. RSVF, respiratory syncytial virus fusion protein. (TIF) [file pbio.3000164.s001.tif]

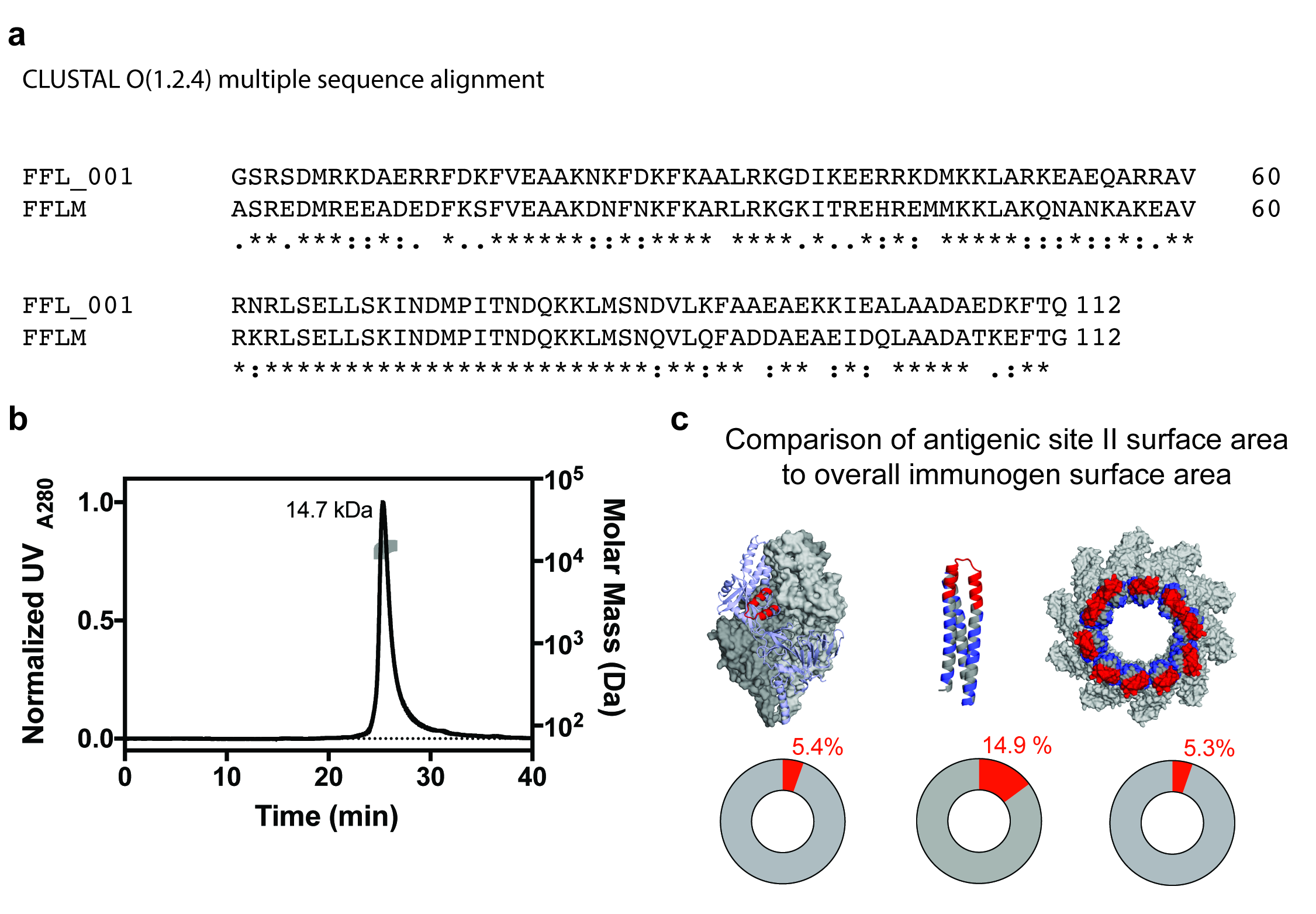

Supplement: S2 Fig — (A) Sequence alignment of FFL_001 and FFLM. (B) Resurfaced variant FFLM is monomeric in solution, as assessed by size exclusion coupled to an online multiangle-light scattering detector. Determined mass in solution is 14.7 kDa ± 3.5%, which is close to the theoretical molecular weight of 14.4 kDa. (C) Relative surface area of RSVF antigenic site II in prefusion RSVF (PDBID 4JHW), FFLM, and NRM (model based on RSVN structure with PDBID 2WJ8). The motavizumab epitope is highlighted in red, blue patches indicate sequence changes of FFLM compared to FFL_001, and pie charts show the fraction of antigenic site II surface area compared to overall immunogen surface area. SASA was computed in PyMol in presence and absence of motavizumab. Percent SASA of antigenic site II is nearly identical when comparing RSVF and NRM, whereas the FFLM monomer shows approximately 3-fold greater relative surface area of antigenic site II, because of its small size. PDB, Protein Data Bank; RSVF, respiratory syncytial virus fusion protein; SASA, solvent accessible surface area. (TIF) [file pbio.3000164.s002.tif]

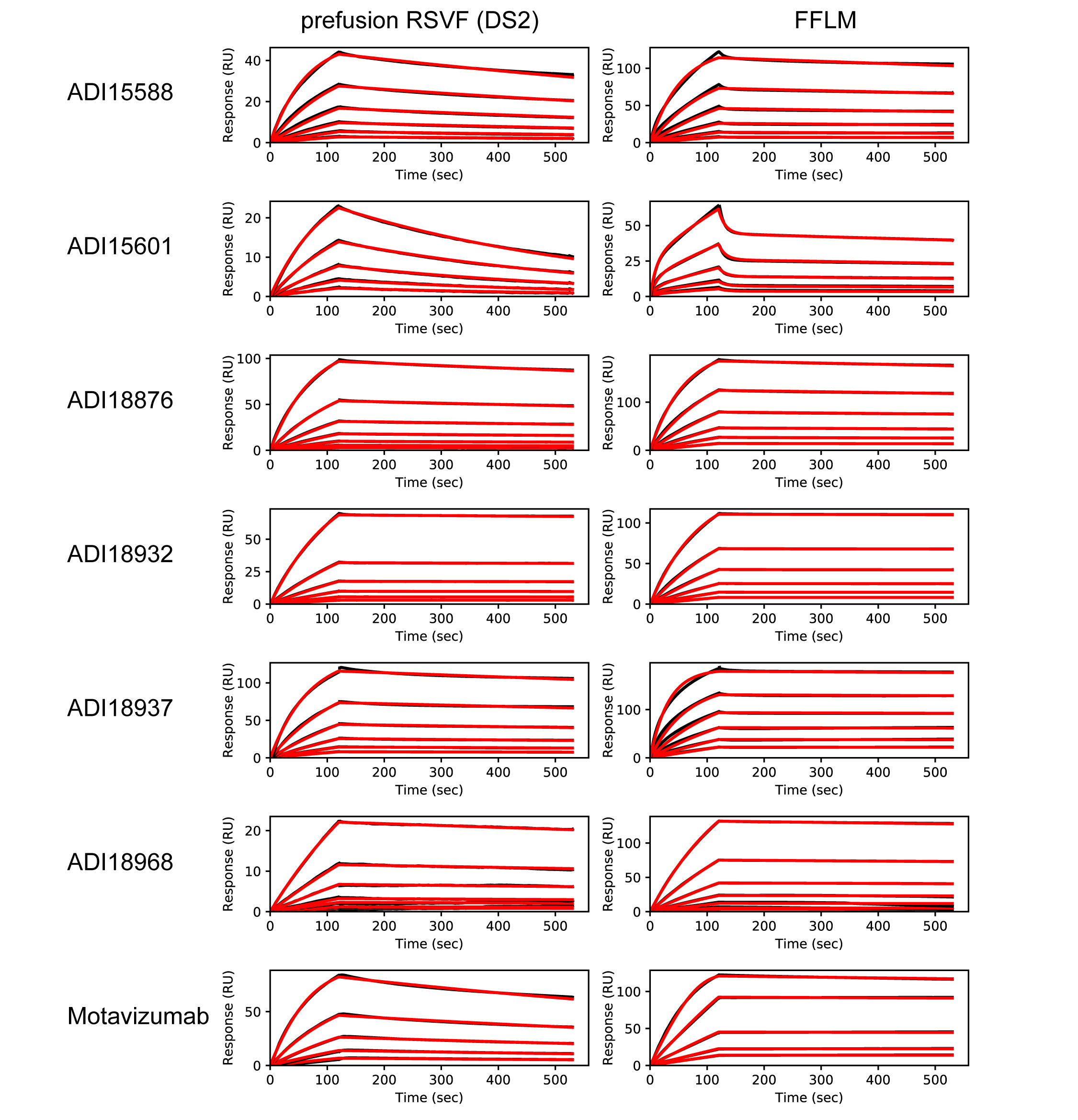

Supplement: S3 Fig — Prefusion RSVF or FFLM was immobilized on the sensor chip surface via amine coupling. Serial dilutions of site II–specific Fabs were injected as analyte. With the exception of ADI15601, which was fitted to a two-state reaction model for binding to FFLM, all data were fitted to a 1:1 Langmuir model within the Biacore evaluation software (GE Healthcare). Fab, antibody variable fragment; nAb, neutralizing antibody; RSVF, respiratory syncytial virus fusion protein; SPR, surface plasmon resonance. (TIF) [file pbio.3000164.s003.tif]

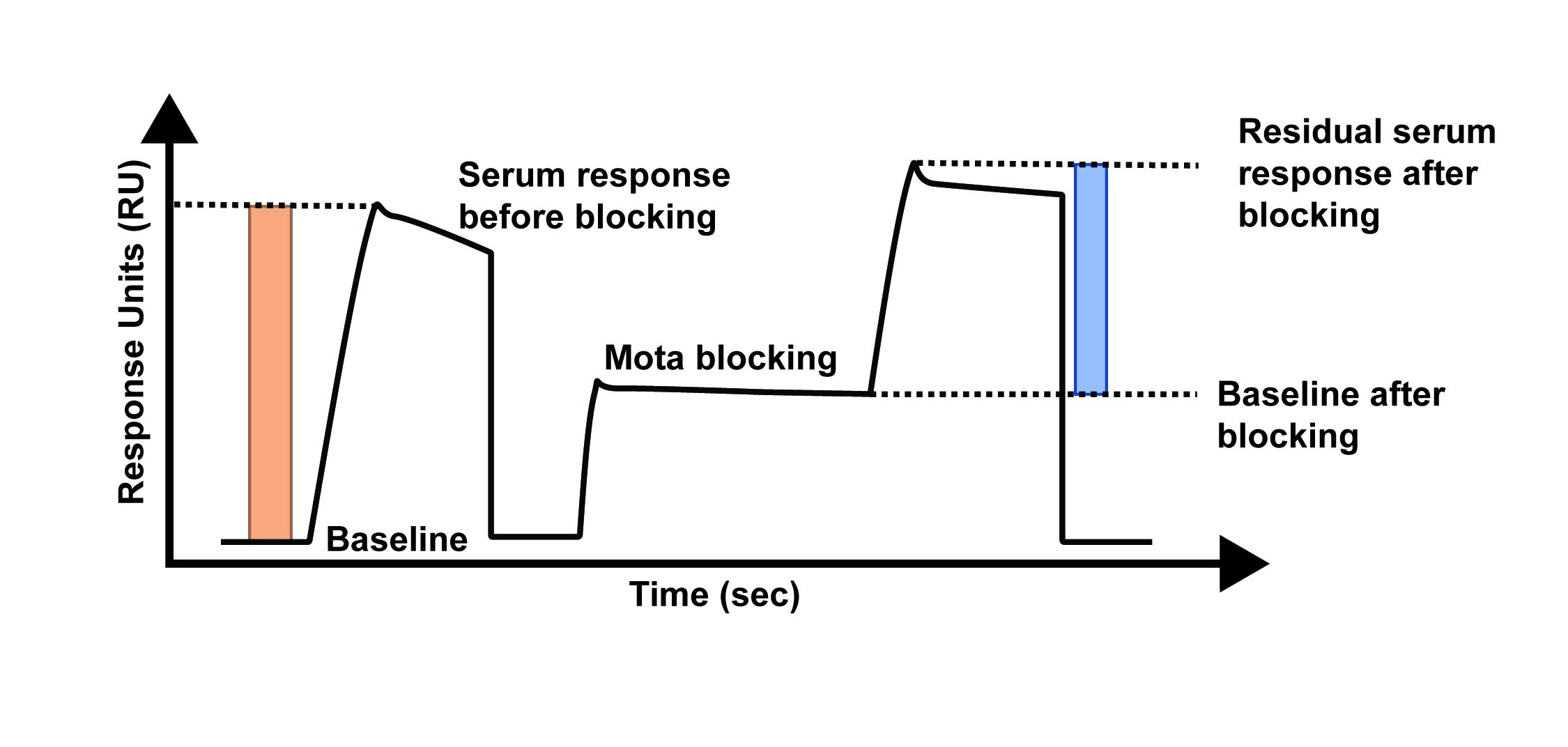

Supplement: S4 Fig — Mouse sera were injected on an antigen-coated sensor chip surface to measure initial response (orange). Following regeneration, motavizumab binding sites were blocked with saturating amounts of motavizumab. Residual serum response was determined on a blocked surface (blue). For data analysis, response units at indicated time points were extracted, and percent competition was calculated as described in Methods and shown in S1 Data. (TIF) [file pbio.3000164.s004.tif]

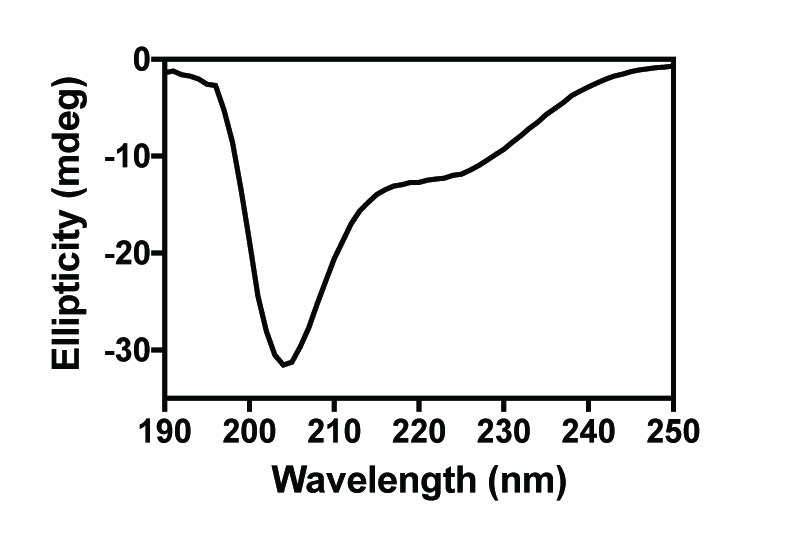

Supplement: S5 Fig — The site II peptide adopts a flexible conformation in solution, measured in phosphate-buffered saline buffer at 25°C. (TIF) [file pbio.3000164.s005.tif]

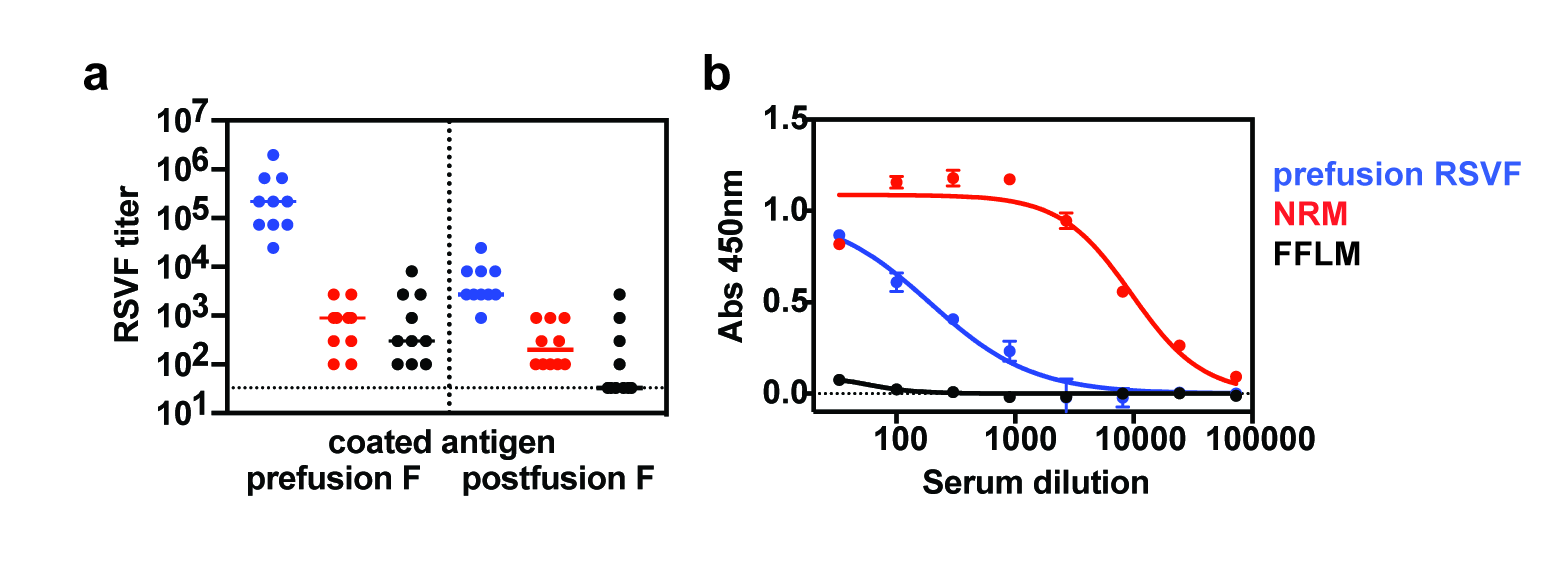

Supplement: S6 Fig — Mice were immunized three times with prefusion RSVF, NRM, or FFLM as shown in Fig 2. (A) Sera from day 56 were analyzed by ELISA for binding to prefusion and postfusion RSVF. Prefusion RSVF–immunized mice showed lower reactivity to postfusion RSVF than to the prefusion form. FFLM- and NRM-immunized mice showed low levels of cross-reactivity with pre- and postfusion RSVF. Data shown are from one out of three independent experiments. (B) Day 56 sera from 10 mice were pooled and tested for binding to lysate of Hep2 cells, which had been infected for 48 hours with RSV. As background control, noninfected Hep2 cell lysate was prepared, and curves shown were background-subtracted. NRM-immunized mouse sera strongly react with viral lysate, whereas mice immunized with FFLM only showed negligible binding to viral lysate. The strong reactivity of NRM-immunized mice derives from antibodies raised against the RSVN carrier protein. Sera from prefusion RSVF–immunized mice are shown as control. Data shown are from one experiment performed in triplicates. Data are available in S1 Data. RSVF, respiratory syncytial virus fusion protein; RSVN, RSV nucleoprotein. (TIF) [file pbio.3000164.s006.tif]

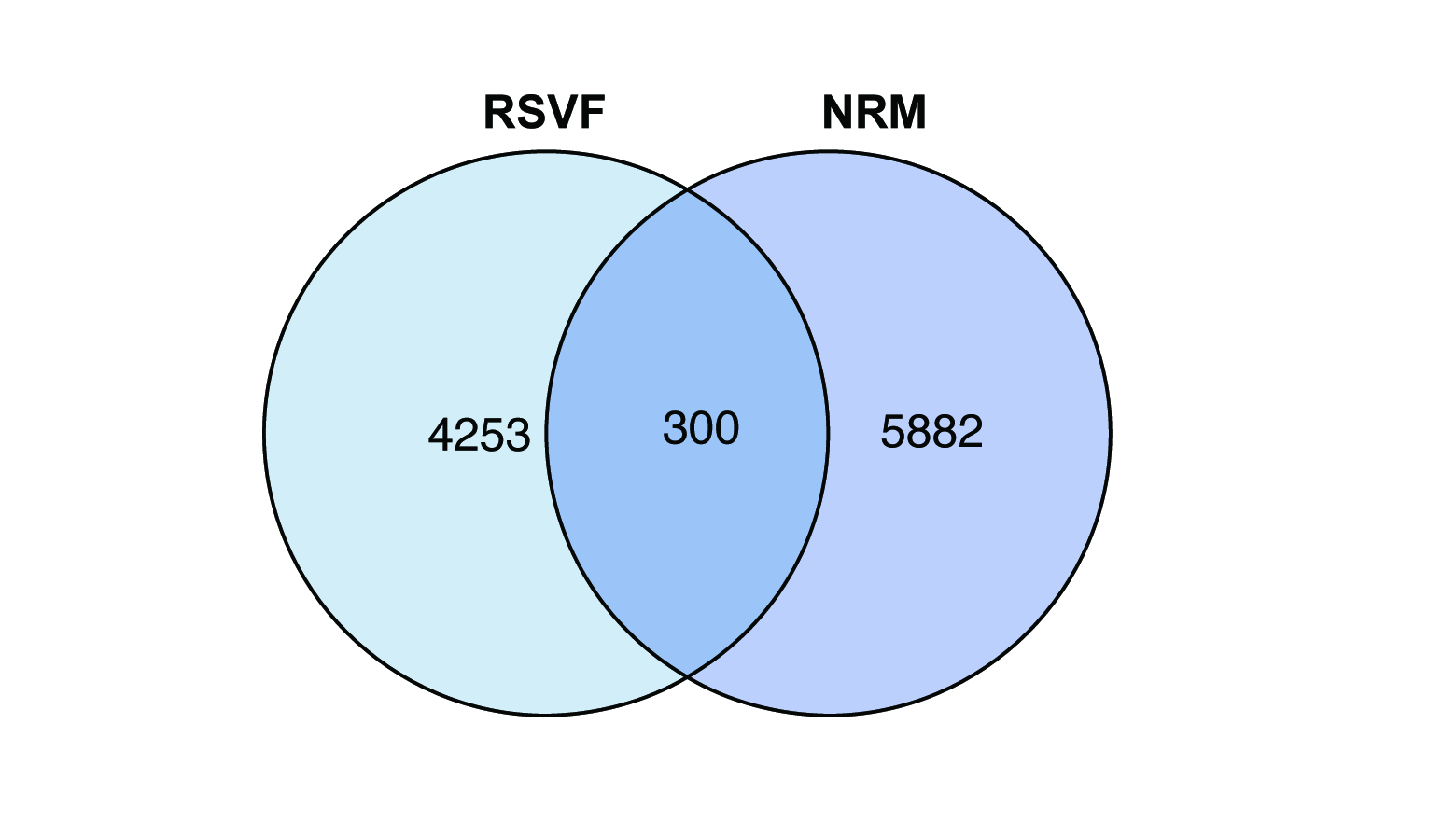

Supplement: S7 Fig — When comparing clonotypes, defined as the same VH gene and 80% sequence similarity in the HCDR3, NRM, and RSVF immunizations yield 300 overlapping clonotypes. HCDR3, heavy chain complementarity-determining region 3; RSVF, respiratory syncytial virus fusion protein. (TIF) [file pbio.3000164.s007.tif]

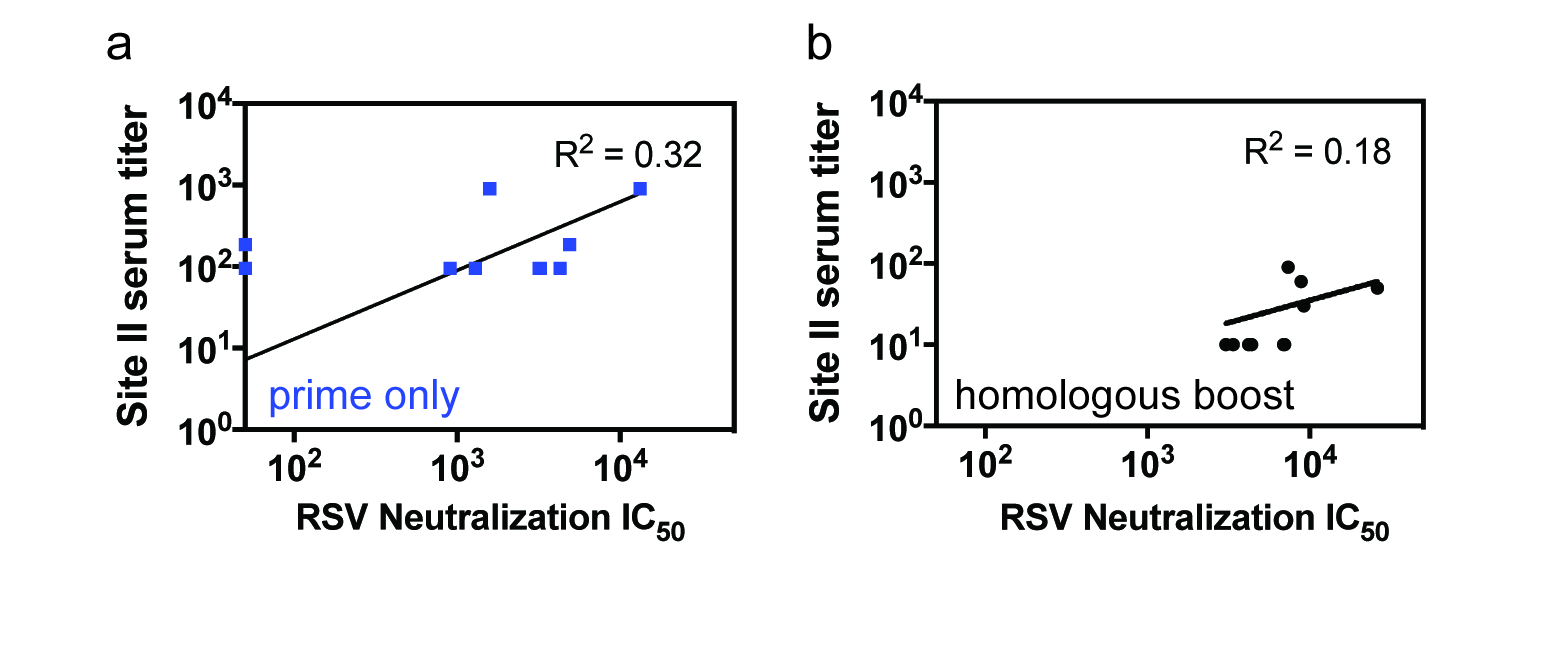

Supplement: S8 Fig — Correlations for the (A) prime-only mouse cohort and the (B) homologous boost cohort. Data represent the mean of two independent experiments, each measured in duplicates. Pearson correlation coefficients (r2) and p-values were calculated in GraphPad Prism. Data are available in S1 Data. RSV, respiratory syncytial virus fusion protein. (TIF) [file pbio.3000164.s008.tif]

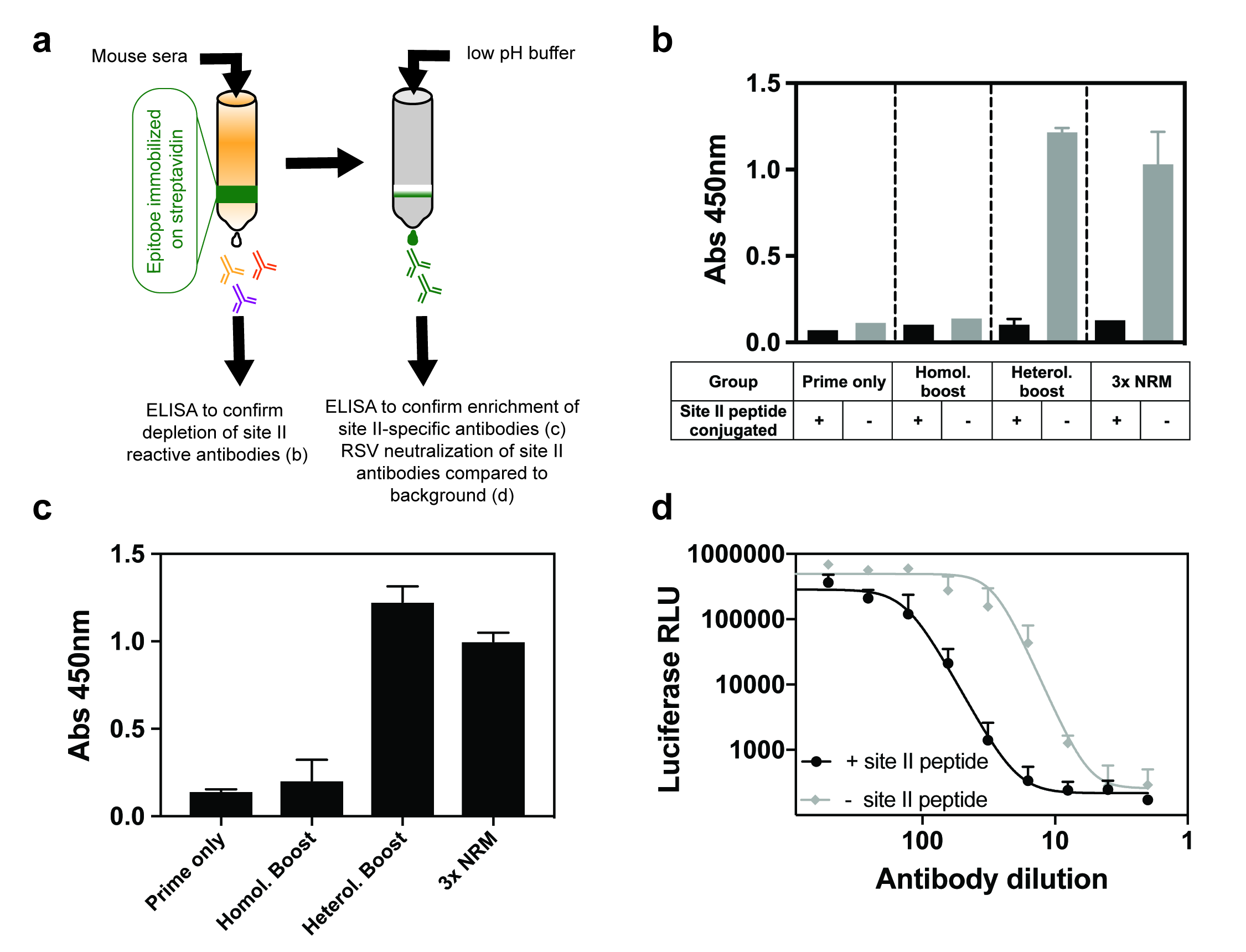

Supplement: S9 Fig — (A) Experimental setup. Streptavidin agarose beads were conjugated to biotinylated antigenic site II peptide. As control, unconjugated streptavidin beads were prepared. Sera from 10 mice within each cohort were pooled and mixed with conjugated and unconjugated beads. Column flow-through and elution fractions were analyzed by ELISA (B, C), and eluted site II–specific antibodies were analyzed in an RSV neutralization assay (D). (B) Analysis of column flow-through for site II peptide reactivity by ELISA. Immunization groups as described in Fig 4 (prime-only, homologous boost, heterologous boost, and 3x NRM). ELISA signal (OD at 450 nm) for site II peptide reactivity is shown for column flow-through from serum fractionation as depicted in (A). Streptavidin beads that were not coupled to antigenic site II peptide were used as controls and did not deplete site II reactivity in the flow-through. Data and error bars presented are averaged from two independent experiments. (C) ELISA against antigenic site II peptide of the elution fractions as shown in (A). Antibodies eluted bound specifically to the antigenic site II peptide. (D) Example RSV neutralization assay curves from elution fractions, obtained from site II conjugated (black) or unconjugated streptavidin beads (gray). Luciferase signal is plotted on the y-axis and is a measure for RSV replication as previously reported [76]. The dilution factor of purified antibodies is indicated on the x-axis. Data shown are from one experiment performed in duplicates. Data are available in S1 Data. RSV, respiratory syncytial virus. (TIF) [file pbio.3000164.s009.tif]

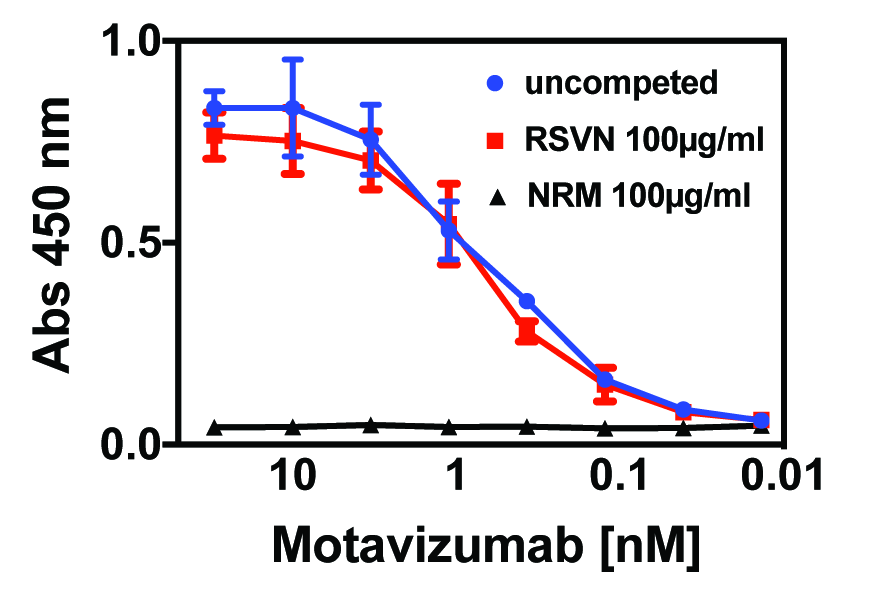

Supplement: S10 Fig — Plates were coated with prefusion RSVF as described in the Methods. Three-fold serial dilutions of motavizumab (initial concentration = 30 nanomolar) were prepared in presence of different competitors (RSVN, NRM, or none). Following overnight competition at 4°C, binding of motavizumab to RSVF was measured. As expected, RSVN competition did not affect RSVF binding of motavizumab. In contrast, NRM efficiently competed with RSVF for motavizumab binding at the indicated competitor concentration. Data shown are from one experiment, with error bars derived from technical duplicates. Data are available in S1 Data. RSVF, respiratory syncytial virus fusion protein. (TIF) [file pbio.3000164.s010.tif]
